# High-content imaging-based BAC-GFP toxicity pathway reporters to assess chemical adversity liabilities

**DOI:** 10.1007/s00204-016-1781-0

**Published:** 2016-06-29

**Authors:** Steven Wink, Steven Hiemstra, Bram Herpers, Bob van de Water

**Affiliations:** Division of Toxicology, Leiden Academic Centre for Drug Research, Leiden University, Einsteinweg 55, 2333 CC Leiden, The Netherlands

**Keywords:** High-content imaging, DILI, Adaptive stress signaling

## Abstract

Adaptive cellular stress responses are paramount in the healthy control of cell and tissue homeostasis and generally activated during toxicity in a chemical-specific manner. Here, we established a platform containing a panel of distinct adaptive stress response reporter cell lines based on BAC-transgenomics GFP tagging in HepG2 cells. Our current panel of eleven BAC-GFP HepG2 reporters together contains (1) upstream sensors, (2) downstream transcription factors and (3) their respective target genes, representing the oxidative stress response pathway (Keap1/Nrf2/Srxn1), the unfolded protein response in the endoplasmic reticulum (Xbp1/Atf4/BiP/Chop) and the DNA damage response (53bp1/p53/p21). Using automated confocal imaging and quantitative single-cell image analysis, we established that all reporters allowed the time-resolved, sensitive and mode-of-action-specific activation of the individual BAC-GFP reporter cell lines as defined by a panel of pathway-specific training compounds. Implementing the temporal pathway activity information increased the discrimination of training compounds. For a set of >30 hepatotoxicants, the induction of Srxn1, BiP, Chop and p21 BAC-GFP reporters correlated strongly with the transcriptional responses observed in cryopreserved primary human hepatocytes. Together, our data indicate that a phenotypic adaptive stress response profiling platform will allow a high throughput and time-resolved classification of chemical-induced stress responses, thus assisting in the future mechanism-based safety assessment of chemicals.

**Electronic supplementary material:**

The online version of this article (doi:10.1007/s00204-016-1781-0) contains supplementary material, which is available to authorized users.

## Introduction

In the past decades, hepatic toxicity has contributed disproportionately to drug withdrawals (Stevens and Baker 2009). Nowadays, drug-induced liver injury (DILI) is still notoriously difficult to predict in as well preclinical and clinical trial settings because of the often idiosyncratic nature. There is a strong incentive to integrate human-relevant mechanistic understanding of adverse drug reactions in in vitro-based data for evidence and read across based on approaches for risk assessment. Transcriptomics has contributed much to our mechanistic understanding and has helped to initiate and populate the adverse outcome pathway (AOP) framework (Ankley et al. 2010; Vinken 2013). AOPs are described as a sequential chain of causally linked events at different levels of biological organization that together culminate in the adverse health outcome. While some AOPs have so far been established, a next important step is to translate AOP-related mechanistic understanding in advanced, preferably quantitative, high throughput assays that reflect pathways essential in target organ toxicity. Our vision is to establish an imaging-based platform that can quantitatively assess the activation of individual key events relevant to AOPs. Our initial focus is on adaptive stress response pathways, which are typically part of AOPs and related to adverse drug reactions.

Chemicals may interact with cellular components, leading to an altered cell biochemical status. Cells sense these biochemical changes and activate specific adaptive stress response pathways. These pathways are activated to combat detrimental conditions under which cells cannot function normally. Classical adaptive stress response pathways are the antioxidant pathways (OSR) mediated by activation of the Nrf2 transcriptional program (Venugopal and Jaiswal 1998), the endoplasmic reticulum (ER) unfolded protein response (UPR) mediated by Xbp1, Atf4 and Atf6 transcription factor activation (Kim et al. 2006), and the DNA damage response (DDR) pathway typically related to activation of the p53 (*TP53*) transcriptional program (Girinsky et al. 1995; Reed et al. 1995). We propose that the quantitative dynamic monitoring of the activation of these adaptive stress response pathways at the single-cell level in high throughput systems will significantly contribute on the hand to chemical safety assessment.

**Fig. 1:**
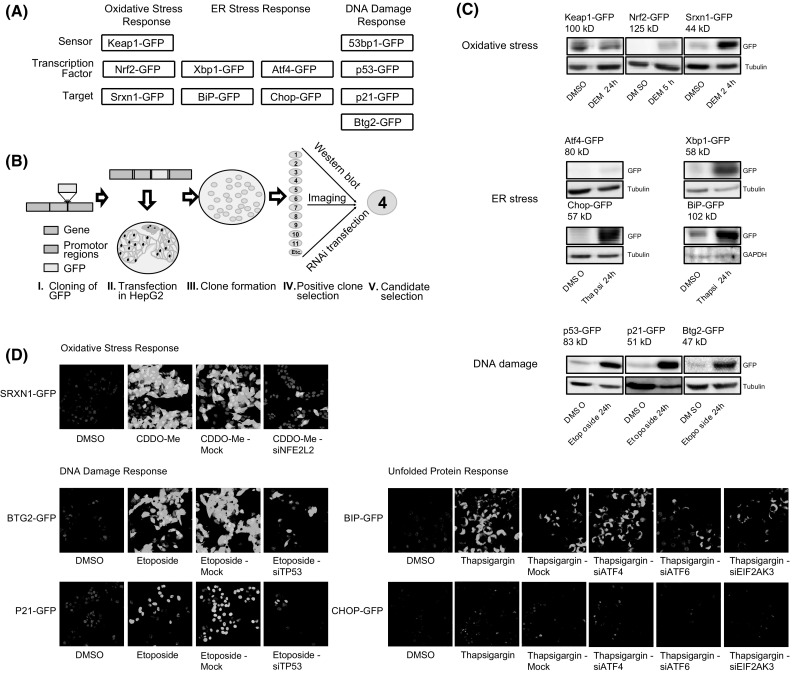
Selection and characterization of adaptive stress response pathway markers for OSR, UPR and DDR. **a** Selection of the individual reporters for the respective pathways representing ‘sensor,’ transcription factor and target genes. **b** Insertion of GFP into BAC plasmid is followed by transfection and selection of the (monoclonal) HepG2 reporter. The selection process involves: (1) imaging of 10–24 transfected HepG2 clones to determine suitability (fluorescence intensity and cell–cell variability) as a reporter cell line, with or without exposure to a stress-inducing compound depending on the reporter type, (2) determining the size of the target protein-GFP fusion and induction level after stress-inducing exposure by Western blot. **c** Western blot analysis of reporter expression under control conditions and treatment conditions. Reporters for oxidative stress (Keap1, Nrf2 & Srxn1), ER-stress (Atf4, Xbp1, Chop & BiP), DNA damage (p53, p21 & Btg2). The size and responsiveness to chemical stress of the GFP-fusion protein product were evaluated. Cells were treated with 100 μM DEM (oxidative stress), 25 μM etoposide (DDR) and 1 μM thapsigargin (UPR) for the either 5 h (Nrf2-GFP) or 24 h (all others) followed by WB analysis. **d** Responsiveness of target genes was assessed by knock down for Nrf2 (Srxn1 activation), p53 (p21 and Btg2 activation) and UPR transcription factors Xbp1, Atf4 or Atf6 (BiP and Chop activation). Mock is the control condition transfected with transfection reagents, but without siRNA

All the above-mentioned adaptive stress response pathways can roughly be conceived as three consecutive steps: (1) ‘sensing’ of the biochemical perturbations; (2) downstream transcription factor activation through either stabilization and/or nuclear translocation; and (3) downstream target gene activation. For the OSR, this involves: (1) Keap1 modulation, (2) Nrf2 stabilization and nuclear translocation, followed by (3) target gene expression including Srxn1 (Herpers et al. 2015; Mazur et al. 2010). The UPR involves (1) sensing of unfolded proteins in the lumen of the ER by BiP, IRE1, PERK and Atf6, followed by (2) downstream transcription factor stabilization and nuclear translocation of Atf4, ATF6 and Xbp1 and (3) subsequent activation of the expression of the chaperone BiP/*GRP78*/*HSP5A* and the transcription factor *DDIT3*/Chop (Takayanagi et al. 2013). Finally, the DDR involves (1) recognition of DNA damage sites and DNA damage foci formation with accumulation of, e.g., 53bp1 in these foci, (2) subsequent stabilization of p53 through phosphorylation by kinases activated after DNA damage and (3) expression key p53 target genes upon translocation of p53 to the nucleus including p21 (*CDKN1A*) and Btg2 (d’Adda di Fagagna et al. 2003; Reinke and Lozano 1997) (see Fig. 1a). We anticipate that the integration of all these different sensors, transcription factors and downstream targets in fluorescent protein reporters would facilitate the evaluation of the dynamic activation of adaptive stress responses at the single-cell level using high-content imaging approaches. Therefore, the aim of the current work was to establish and systematically evaluate the application of GFP reporters using HepG2 cell lines for these three pivotal adaptive stress response pathways using bacterial artificial chromosome (BAC) cloning technology (Poser et al. 2008b), targeting individual ‘sensor’ proteins, transcription factors as well as downstream target proteins. Since DILI prediction remains a major problem, we focused on the integration of these reporters in the liver hepatoma cell line HepG2, which is routinely used for high throughput first tier liver toxicity liability assessment (Knasmuller et al. 2004; Lin and Will 2012; Maness et al. 1998).

Here, we established, characterized and evaluated in total eleven BAC-GFP HepG2 reporter cell lines reflecting three adaptive stress response pathways for the application in live cell high-content imaging in relation to a set of DILI reference compounds. Our data indicate that these reporter cell lines consistently and selectively monitor the dynamic activation of the OSR, UPR and DDR at the single-cell level for pathway-specific compounds. Moreover, when we correlate the HepG2 BAC-GFP with activation of adaptive stress response in primary human hepatocytes we are able to identify the activation of these stress response pathways that are typically seen by DILI drugs in primary human hepatocytes. Interestingly, the live cell acquisition data allow the improved classification of DILI compounds based on dynamic stress pathway activation.

## Results

### GFP-tagged stress-reporter proteins respond to corresponding chemically induced stress

To enable live cell imaging of the chemically induced dynamics of cellular adaptive stress response programs, a panel of reporter cell lines was created using BAC cloning technology (Poser et al. 2008a). For each adaptive stress response pathway, an upstream ‘sensor,’ a transcription factor and a downstream target were chosen (Fig. 1a). For the oxidative stress response program (OSR), kelch-like ECH-associated protein 1 (Keap1) was selected as upstream sensor, nuclear factor, erythroid 2-like 2 (Nrf2/*NFE2L2*) as transcription factor and Srxn1 as downstream target (Herpers et al. 2015; Itoh et al. 2004). For the UPR, heat shock 70 kDa protein 5 (BiP/*HSPA5*) regulates the endoplasmic reticulum (ER)-stress/unfolded protein response (UPR) pathway through binding to accumulated unfolded proteins and consequently dissociating from the transmembrane transducers Atf6, PERK and IRE-1 (Hetz et al. 2015); as such, BiP acts as a sensor of the UPR. However, BiP is also induced strongly after ER-stress (Gulow et al. 2002) and also reflects UPR activation. We labeled two arms of the UPR: For the pro-survival route, we labeled the transcription factor Xbp1 and downstream target chaperone BiP; and for the translation inhibition and pro-apoptotic arm, we labeled the activating transcription factor 4 (Atf4) and DNA-damage-inducible transcript 3 (*DDIT3*/Chop). For the DNA damage response program (DDR), the upstream sensor tumor protein p53-binding protein 1 (TP53BP1/53bp1) was chosen based on its ability to sense double-strand breaks (Lee et al. 2014) and activate the ataxia telangiectasia-mutated protein pathway (ATM). For the DDR, tumor protein p53 (*TP53*/p53) was chosen as the pivotal transcription factor; finally, the two p53 downstream targets cyclin-dependent kinase inhibitor 1 (*CDKN1A*/p21) and BTG family member 2 (Btg2) were selected. To ensure near-endogenous protein-fusion levels and normal regulation of these adaptive stress response programs, enhanced green fluorescent protein (eGFP) and selection markers were cloned in bacterial artificial chromosome (BAC) vectors, which consist of genomic DNA which still contain the endogenous promoter, enhancers and introns. BACs were selected that contained at least 10 kbp on either side of the exon domains.

The BAC-GFP constructs were created using homologues recombination with pRed/ET recombinase, and these constructs were used to transfect HepG2 as described previously (Hendriks et al. 2012). Viable HepG2 colonies were passaged separately to obtain monoclonal BAC-GFP cell lines. For each target gene, a single monoclonal BAC-GFP cell line was selected based on fluorescent intensity and protein size (Fig. 1b). All selected reporter lines were evaluated on fusion protein size, responsiveness to selective pathway activators and targeted knock down by RNAi (Fig. 1c, d). The GFP-tagged protein sizes for all targets with the exception of Nrf2 [which runs at 95 kDa instead of the theoretical 67 kDa as reported previously (Lau et al. 2013)] were in line with reported values (http://www.genecards.org/). While Keap1-GFP levels were not induced by the pro-oxidant DEM, as expected, the levels of Nrf2-GFP and Srxn1-GFP were clearly induced by DEM. The ER-stress reporters Atf4-GFP, Chop-GFP, Xbp1-GFP and BiP-GFP clearly responded to the ER-stress inducer thapsigargin. The DDR reporters p53-GFP, p21-GFP and Btg2-GFP are clearly induced after 24-h exposure of the topoisomerase inhibitor etoposide; the large size of 53bp1-GFP (241 kDa) prohibited qualitative assessment by Western blotting.

**Fig. 2:**
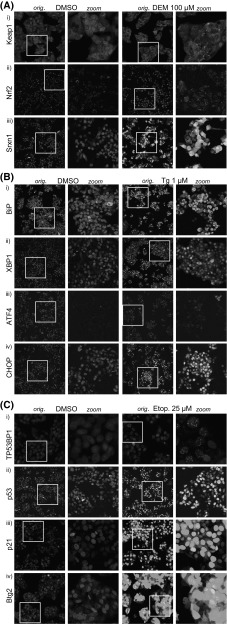
Representative confocal images of BAC-GFP adaptive stress response reporters. Representative confocal images are shown for OSR: Keap1, Nrf2 and Srxn1 (**a**); UPR: BiP, Xbp1, Atf4 and Chop (**b**), and DDR: 53bp1, p53, p21 and Btg2 (**c**). Two left columns reflect vehicle treatment for 24 or 5 h for Nrf2 (left column overall image; right column zoomed image); the two right panels reflect model compound treatment for 24 h or 5 h for Nrf2 (left column overall image; right column zoomed image): OSR, 100 μM DEM; UPR, 1 μM thapsigargin; DDR, 25 μM etoposide. Images of most reporters are captured at 20 or 40 times magnification on 512 × 512 pixels; however, the reporters Keap1 and 53bp1 require a higher resolution to be able to count the number of foci per cell, and as such these were captured at 40× magnification on 1024 × 1024 pixels. Hoechst channel is omitted for low intensity-level reporters in the right columns (zoom) panel

Cellular localization of GFP-fusion products for all reporters was evaluated by confocal microscopy for control and compound treatment for 5 h (Nrf2) or 24 h (all others) (Fig. 2). A clear increase in levels of all downstream targets Srxn1-GFP, Btg2-GFP and BiP-GFP in the cytosol was seen. For the transcription factors Nrf2-GFP, Xbp1-GFP, Chop-GFP and p53-GFP as well as p21-GFP, an increase in nuclear intensity was observed. An increase in the number of nuclear DNA damage foci for 53bp1-GFP and cytosolic autophagosome-related foci for Keap1-GFP is also evident (autophagosomes co-localizes with p62 in immunofluorescent experiments, indicating autophagosomal location of Keap1-GFP (data not shown)). Little increase in Atf4-GFP was visible, yet image analysis revealed a clear and selective increase (see later Fig. 4).

**Fig. 3:**
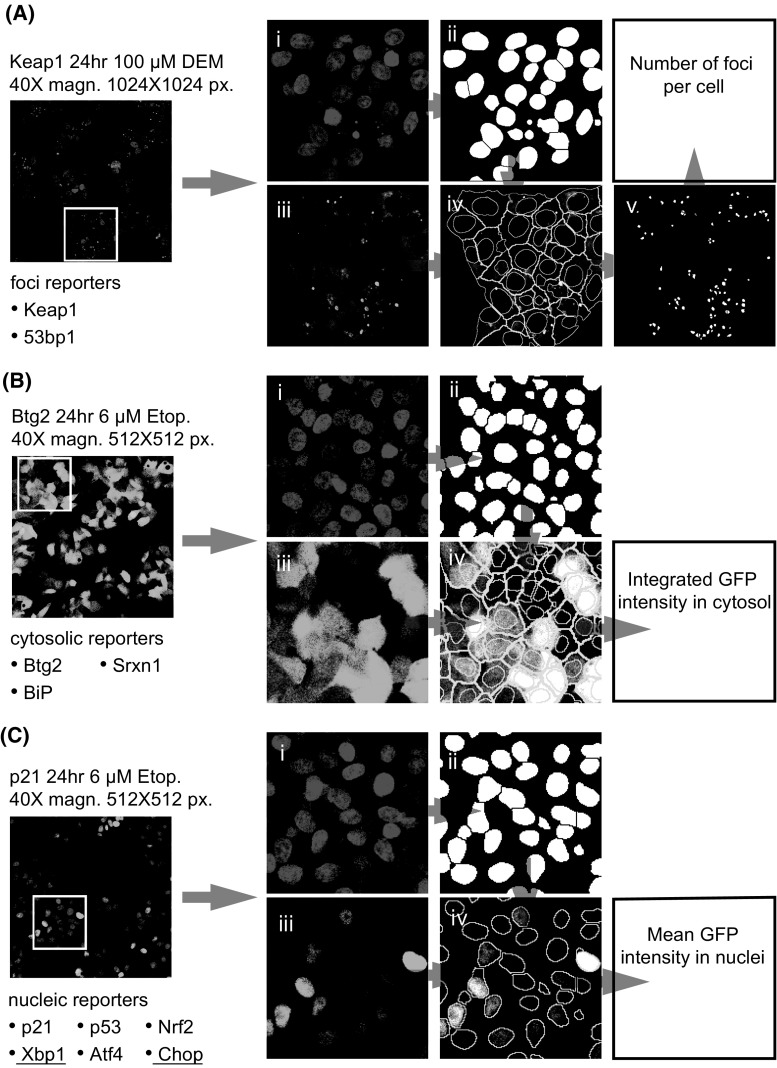
Automated image analysis of BAC-GFP reporter cell lines. Automated imaged analysis was performed using CellProfiler and ImageJ-based algorithms as described in “Materials and methods” section. **a** The Keap1 and 53bp1 reporters were based on foci detection. Left panel: A 1024 × 1024 pixel 40 times magnified image of Keap1-GFP reporter after 24-h exposure to 100 μM DEM. *Blue* staining corresponds to the nuclei (*i*) and *green* corresponds to the Keap1-GFP-fusion protein (*iii*). The nuclei are segmented (*ii*) and used as seeds for the cytosol identification using the GFP signal (*iv*), the outlines of the nuclei and cytosols are displayed as *yellow lines*. Next, the GFP-signal foci corresponding to Keap1-GFP being degraded in autophagosomes are segmented (*v*) and assigned to individual cells. **b** The Btg2, Srxn1 and BiP reporters are based on quantifying the GFP signal in the cytosolic region of cells. First, the nuclei signal (*i*) is segmented (*ii*) and used as seeds for the cytosol identification (*iii*, *iv*). **c** The p21, p53, Nrf2, Xbp1, Atf4 and Chop reporters are based on quantifying the GFP signal in the nuclei. The nuclei signal (*i*) is segmented (*ii*), and these regions (*iv*) are directly used to quantify the GFP intensity (*iii*) (color figure online)

Next for all individual BAC-GFP reporters, an automated multi-parameter imaging analysis pipeline was established using CellProfiler (Kamentsky et al. 2011) software and ImageJ plug-ins (Fig. 3). Depending on the BAC-GFP reporter type, the different imaging readouts were determined using automated image analysis. For 53bp1-GFP and Keap1-GFP, we quantified foci formation in the cytosolic (Keap1-GFP translocation with autophagosomes) and nuclear compartment (53bp1-GFP localization in DNA damage foci), respectively. For Srxn1, BiP and Btg2, we quantified the integrated GFP intensity in the cytosol. For Nrf2, Xbp1, Atf4, Chop, p53 and p21, we determined the mean GFP intensity in the nucleus. The different quantitative measurements reflect the altered expression and localization of our stress reporters.

Altogether, we have established a functional panel of adaptive stress response reporters that allow us to quantitatively assess the dynamic activation of individual pathway components in living cells at the single-cell level population level.

### Adaptive stress response BAC-GFP reporters respond in a sensitive and selective manner to reference compounds

As a next step, we set out to test the responsiveness and selectivity of the panel of stress-reporter cell lines to: (1) oxidative stress-inducing agents DEM, CDDO-Met [a pharmacological inducer of Nrf2 activity, (Yang et al. 2009)] and iodoacetamide (IAA); (2) DNA-damage-inducing agents etoposide and cisplatin; and (3) UPR-inducing agents brefeldin A (BFA), tunicamycin (Tc) and thapsigargin (Tg) (Supplemental Table 1). To monitor signaling programs well before any significant cytotoxicity occurs and, thereby, deduce causative relationships for the onset of cytotoxicity, compound concentrations were chosen that would not lead to significant cell death after 24 h as well as two additional concentrations that were twofold and fourfold lower to assess the overall sensitivity of the reporter panel. Reporter cell lines were imaged for a period of 24 h using live cell confocal imaging and evaluated for onset of cytotoxicity by propidium iodide (PI) exclusion (Supplemental Fig. 1). Little cell death was observed, and no major differences between cell lines were discernable.

**Fig. 4:**
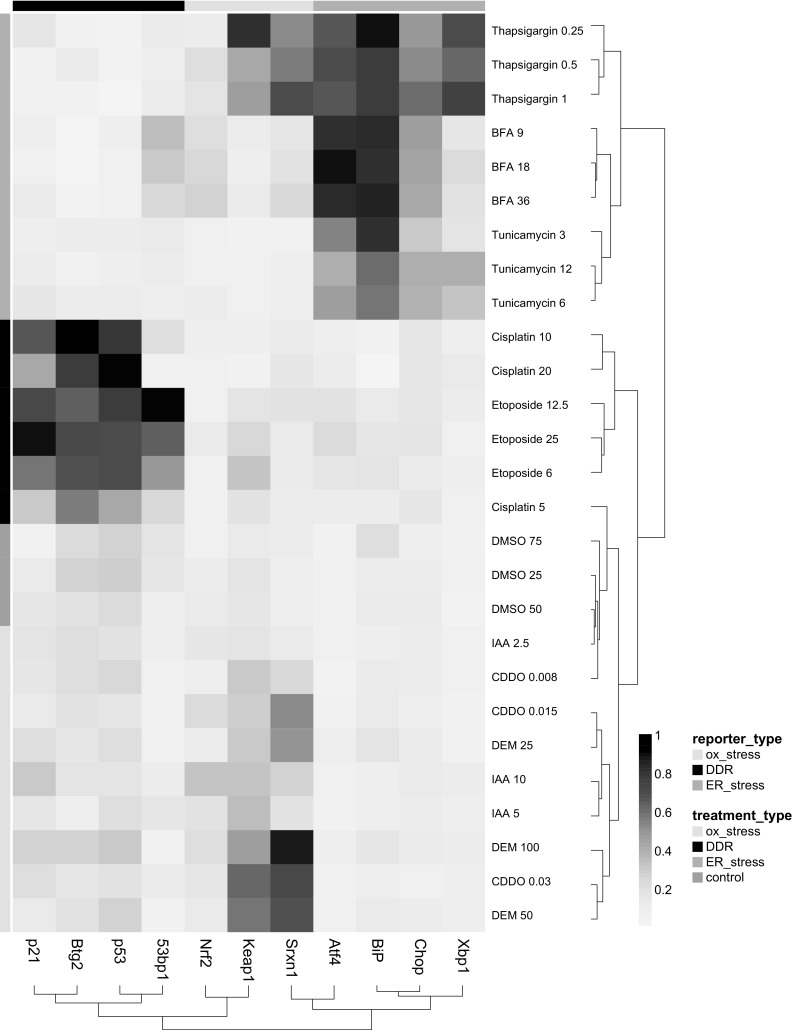
Effect of reference compounds on adaptive stress GFP reporter response. Heatmap displays the individual GFP reporter and compound measurements of the various reference compounds in all reporter cell lines. Shown are the 24-h endpoint measurements as the average of three independent experiments. *Color* intensity corresponds to plate-cell line-normalized feature values. Data shown were subjected to unsupervised hierarchical clustering. *Side bars* correspond to stress pathway reporter type (*top bar*) and reference compound treatment class (*side bar*) (color figure online)

We set out to obtain mechanistic information on the mode of activation of our different reporters and anticipated a selective activation by our reference compounds. We first evaluated whether, as a simplified method, only the final time point of the live imaging dataset would be sufficient to determine reporter activation. The endpoints from the different quantitative features of each reporter (see Fig. 3) were collected for each reference compound concentration range and subjected to an unsupervised hierarchical clustering (Pearson distance method and Ward clustering) and displayed as a heatmap (Fig. 4). The heatmap showed a clear clustering of the reporter cell lines and reference compound groups within the corresponding adaptive stress response pathway. This was reflected by a significant activation of the GFP reporters. Intriguingly, at this 24-h time point Nrf2-GFP did not show enhanced nuclear localization and for any of the reference compounds, possibly related to an earlier activation. The DNA damage and UPR reporters were all activated by their corresponding reference compound sets. Interestingly, the UPR reference compound thapsigargin also strongly activated the oxidative stress reporters Keap1 and Srxn1, in accordance with observations in neuronal cells (Li and Hu 2015), yet brefeldin A and tunicamycin selectively induced the UPR response. Brefeldin A slightly activated the 53bp1-GFP reporter, while the p53-GFP, Btg2-GFP and p21-GFP were not activated. This underscores the possibility to identify compound-specific responses.

### Live cell imaging of HepG2 reporters defines temporal ranked adaptive stress response profile

**Fig. 5:**
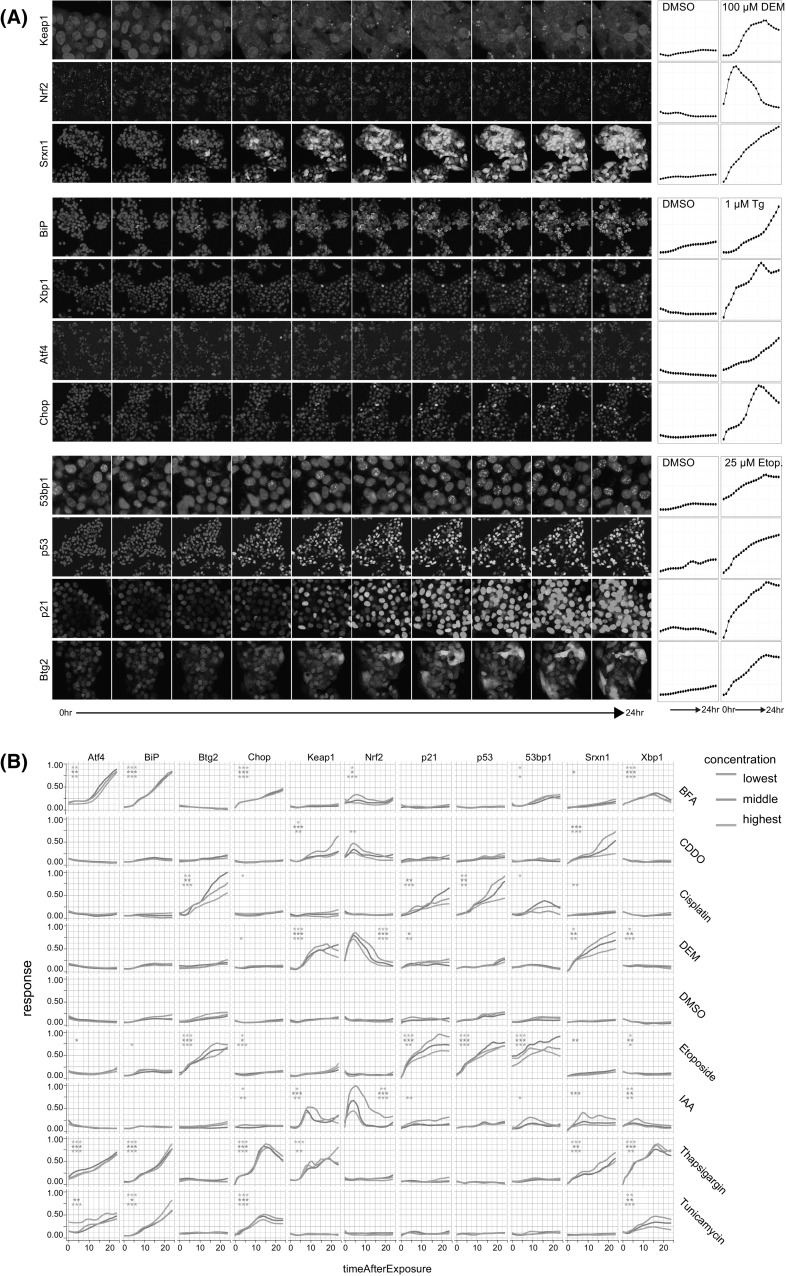
Dynamic GFP reporter activation for different adaptive stress response pathways. **a** Representative images of the dynamic activation of the various stress response pathway reporter cell lines by different reference model compounds: OSR, DEM; UPR, Tg; DDR, Etop). **b** Time dynamics of all reference compounds on the different stress response reporters. Data shown are the normalized values for individual reporters. *Different colors* indicate low (*red*), medium (*green*) and high (*blue*) concentrations. Significance is depicted as *p < 0.05, **p < 0.01 and *** *p* < 0.001 (color figure online)

**Fig. 6:**
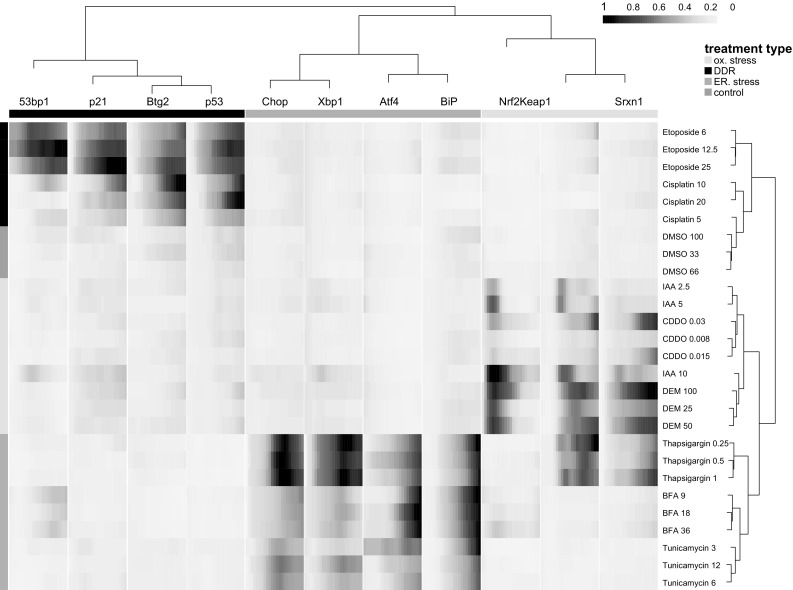
Cubic hierarchical clustering of the time courses of the reporter panel and reference compounds. Time dynamics of all reference compounds on the different stress response reporters was used for cubic hierarchical clustering as described in “Materials and methods” section. Data shown are the normalized values for individual reporters of >3 independent experiments

We obtained detailed live cell imaging data over a 24-h time course for the entire reference dataset. Next, we investigated whether live cell imaging adds value in quantifying adaptive stress response programs. For most reference compounds, reporter activation occurred within the first hours after treatment, dependent on the reporter (Fig. 5). Also, the dynamics of the response differed per reference compound and reporter. Thus, the live cell data demonstrate a rapid accumulation of Nrf2-GFP starting around 2 h and returning to close to baseline levels after 15 h for CDDO-Me, DEM as well as IAA (Fig. 5). IAA exposure caused early activation of several adaptive stress response programs: the OSR reporters Keap1, Nrf2 and Srxn1 but also UPR reporter Xbp1 and DDR reporter 53bp1. Interestingly, while thapsigargin showed strong activation of all UPR reporters as well as the Keap1 and Srxn1 reporter, no clear stabilization of Nrf2-GFP was observed. Next, the entire set of quantitative time course data of the reference compounds for all reporters was subjected to cubic hierarchical clustering (maximum distance measure and complete linkage clustering), thus taking into consideration the time dynamics of each reporter–treatment combination. The reporter and treatment stress types again cluster fully together (Fig. 6). However, by inclusion of the time dynamics into the clustering algorithm compounds with similar time dynamics cluster together within the reference and model compound blocks and thus reveals distinct response-type sub-clusters. This is most evident as the different compounds induce responses with distinct time dynamics, and therefore, the concentration ranges for each compound cluster together, in contrast to the endpoint clustering of Fig. 4. Altogether, this supports the notion that the entire time course dynamics of compound responses on reporter cell lines provides added value for classification of compounds.

### DILI compounds mainly activate OSR and UPR reporter genes in primary human hepatocytes (PHH)

**Fig. 7:**
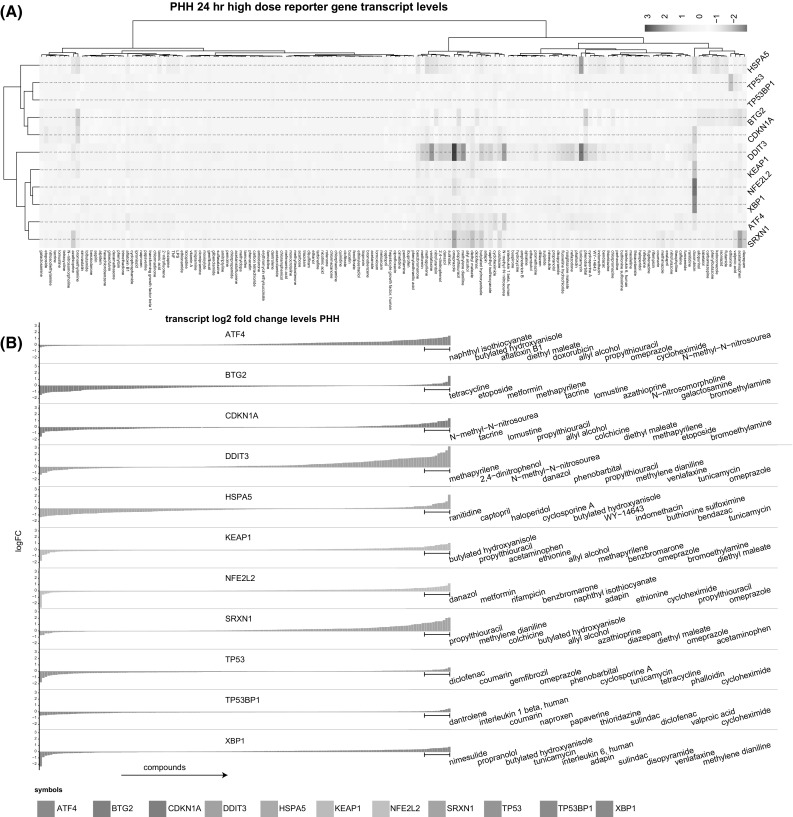
Primary human hepatocyte data from TG-GATES. **a** Unsupervised hierarchical clustering of log2 fold-change values of primary human hepatocyte transcripts in PHH. **b** Rank-ordered transcript fold changes for each reporter gene. Top 10 upregulated compounds per reporter gene are displayed on the *right*

As a next step, we set out to test the reporter platform in a more DILI-relevant setting. To assess the correlation between adaptive stress pathway activation in PHH and that observed in our BAC-GFP reporters, we decided to focus on four downstream targets that showed the most prominent responses in PHH: OSR, Srxn1; UPR, Chop and BiP; DDR, p21. First, we calculated the log2 fold changes for all DILI compounds from the PHH data from the TG-GATES dataset for all our 11 reporter genes. We next subjected these data to hierarchical clustering (Fig. 7a). The oxidative stress transcript levels were increased by a set of 39 compounds with *NFE2L2*, *KEAP1* and *SRXN1* correlation over all treatments being high (Pearson correlation *KEAP1*-*NFE2L2* 0.64, *KEAP1*-*SRXN1* 0.58, *NFE2L2*-*SRXN1* 0.45). The transcript-level responses of the UPR genes were diverse; 53 compounds activated the *DDIT3* of which 33 (62 %) negatively regulated *HSPA5*. *ATF4* seemed to slightly correlate with oxidative stress (Pearson correlation *SRXN1*-*ATF4* 0.4). Hardly any changes in transcript levels of *XBP1* were seen likely due to its mechanism of posttranscriptional regulation (Yoshida 2007).

A very small number of DILI compounds affected *TP53*, *TP53BP1*, *CDKN1A* or *BTG2* transcript levels in PHH; this reflects the thorough screening for DNA damage effects of pharmaceuticals. A small cluster of compounds activated the *CDKN1A* and *BTG2* expression but not *TP53* and *TP53BP1*.

To select a set of DILI compounds that selectively affects individual reporters, we rank-ordered the PHH fold-change transcript-level data for Srxn1, Chop, BiP and p21 and selected 10 compounds that originate from the top 12 ranked compounds as DILI-compound test set (Fig. 7b), in total resulting in 29 different DILI compounds that partly had overlap between downstream targets.

### HepG2 reporters define temporal ranked adaptive stress response profiles of DILI-relevant compounds

**Fig. 8:**
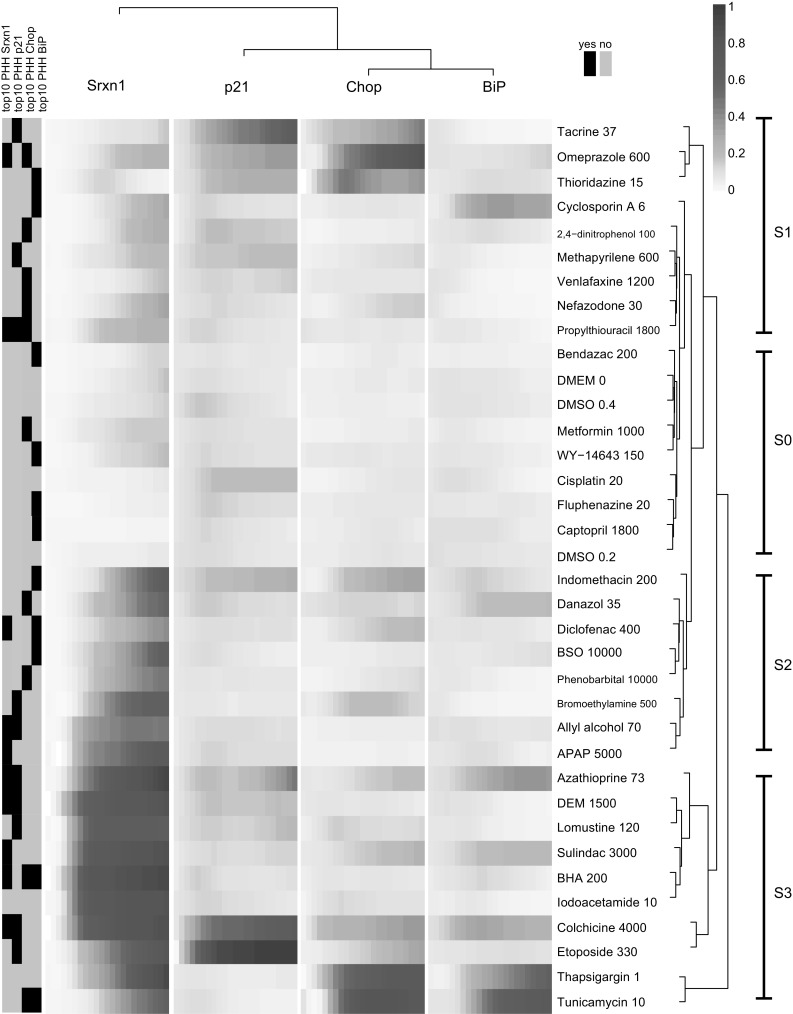
Effect of selected DILI test compounds on stress response activation. DILI compound selection origin is labeled black (*left legend*), 24-h time course corresponds to the 4 individual columns, each column representing a time course for 1 of 4 reporter cell lines. Response magnitude is labeled as *orange* intensity and according to the legend (*top right*). Compounds and concentrations are displayed as rows and labeled on the *right*. The time course profiles were subjected to cubic clustering as described in “Materials and methods” section (color figure online)

Next, we tested the 29 DILI compounds in the Srxn1-GFP, Chop-GFP, BiP-GFP and p21-GFP cell line. For comparative purposes, the same concentrations were used as in the PHH TG-GATES high-dose data. All four reporter cell lines were imaged live for 24 h (Supplemental Fig. 2). The resultant reporter–response time courses were subjected to the same cubic hierarchical clustering, which led to several distinct clusters of response types (Fig. 8). Different response types were defined based on the intensity of the response, the response type and the order of the response types. Based on the Srxn1-intensity level, clusters of no induction (S-0), weak induction (S-1), middle induction (S-2) and strong induction (S-3) can be defined. The S-0 group of compounds includes a set of 7 treatments, which are negative among all 4 reporters. The remaining S-0 treatments showed a weak p21 activation. The S-1 cluster of slightly increased Srxn1 levels is preceded by p21 activation, and in the case of cyclosporin A BiP-GFP levels increased markedly in time preceding Srxn1-GFP activation. Within the strong Srxn1 activation cluster (S-3), a subset of treatments oxidative stress co-occurred with p21 as well, most notably etoposide and colchicine. A distinct adaptive stress response profile was related to strong Chop-GFP induction by tacrine, omeprazole and thioridazine. However, no increase in BiP-GFP chaperone is evident, in contrast to azathioprine and sulindac, which have a low Chop-GFP activation, but a strong BiP-GFP activation. Finally, we assessed the positive co-occurrence of reporter gene activation between reporter transcript levels in PHH and GFP reporter levels in the four reporter cell lines. The correlation was 9/10 for Srxn1-GFP, 6/10 for p21-GFP and 2/10 for Chop-GFP and 2/10 for BiP-GFP (Supplementary Fig. 2).

## Discussion

In the current study, we established a panel of fluorescent protein reporter HepG2 cell lines using BAC cloning technology to follow the dynamics of several adaptive stress response pathways essential in chemical-induced cytotoxicity. We focused on target genes that are central in the regulation of three key adaptive stress response programs; for each pathway, we successfully established reporters for the sensory machinery, downstream transcription factor and one of the transcription factors downstream targets. Our results show that the adaptive stress response reporters are selective and sensitive to their corresponding reference training compounds. Moreover, live cell imaging enabled us to define the temporal order of activation of the adaptive stress response programs initiated after chemical exposure. Furthermore, DILI-related compounds that are strong inducers of our selected adaptive stress response pathways in PHH were positively identified in the HepG2 reporter cell models with Srxn1, Chop and p21.

Monitoring of adaptive stress response pathways as a predictive tool for chemical safety prediction has gained considerable attention in systems toxicology (Jennings et al. 2013; Wink et al. 2014). So far the approaches have largely been used as transcriptomic-based strategies (Jennings et al. 2013; Limonciel et al. 2015). Transcriptomics provides a comprehensive analysis to monitor cellular stress responses to chemicals at a single time point and average population level. The application of our GFP-based reporter cell lines as presented here, in conjunction with high-content live cell imaging, provides various advancements in chemical safety assessment that are not feasible with and/or complementary to transcriptomics. Firstly, we now can quantitatively assess the regulation of the entire adaptive stress response pathway irrespective of transcriptional regulation. Thus, we can monitor the modulation of upstream regulators such as Keap1 and 53bp1, which are constitutively expressed and translocate to the autophagosomes and DNA damage foci, respectively, upon activation. Moreover, we can observe posttranslational regulation of reporter expression of in particular transcription factors due to protein stabilization, e.g., Nrf2, or p53, or alternative processing of mRNA (e.g., Xbp1). Secondly, our GFP-based reporters allow a more mechanistic evaluation of the relationship between stress pathway activation and cytotoxicity since we can follow the onset of stress responses at the real protein expression level, the cell physiology-relevant molecules in cells, in single cells with the subsequent assessment of cell viability (e.g., onset of necrosis or apoptosis). Thirdly, it is more cost- and technically feasible to monitor the response in a high time resolution to determine temporal orders of stress pathway activation. It is of critical importance to define the detailed oscillatory dynamics from, e.g., NF-κB (Fredriksson et al. 2014) that are generally controlled by genetically defined negative feedback loops. Fourthly, the GFP reporters allow the possibility to assess the overall cell and cell organelle morphological perturbations as well as foci formation from, e.g., autophagosomes or DDR repair foci (Wink et al. 2014).

In comparison with previous high-content imaging studies, to our knowledge we developed the first high-content imaging assay able to monitor the response of cells to chemical exposure on a signaling level. Previous high-content imaging studies were based on either cytotoxic parameters such as cell death, ROS, mitochondrial potential and Ca^2+^-based dyes which measure toxic outcome measures and not the cellular responses that combat these adversities, or morphological features which capture morphological changes of cells or organelles and correlate these indirectly to mechanisms or classify morphology-based perturbations due to chemical exposure with the use of training data (Garside et al. 2014; Loo et al. 2007; Persson et al. 2013).

Our data indicate that our BAC-GFP-based reporter approach can clearly reveal subtle differences in the mode of action of compounds. Our UPR reference compounds thapsigargin and tunicamycin both induced the onset of two key UPR reporters, e.g., Chop-GFP and BiP-GFP, to a similar extent and with a similar temporal profile (see Fig. 5). Yet, while thapsigargin also induced a strong induction of the Srxn1 reporter, tunicamycin did not. Thapsigargin causes ER-stress due to its inhibition of the SARC/ER Ca^2+^ ATPase, thereby lowering Ca^2+^ levels in the lumen of the ER. Tunicamycin blocks protein glycosylation in the ER. While both conditions initiate the UPR response, ER calcium perturbations also induce an oxidative stress response. Yet, the latter response is different from compounds that directly target protein thiols, including iodoacetamide and DEM; although thapsigargin caused Keap1-GFP foci formation, this was not associated with a strong accumulation of Nrf2-GFP, which is observed with iodoacetamide and DEM. These results clearly illustrate the strength of the temporal single-cell live cell analysis of adaptive stress responses for mode-of-action clarification. Likewise, such reporter systems may also contribute to the adverse outcome pathway (AOP) toolbox and as such quantify the activation of individual key events that reflect and are critical in toxicological-relevant AOPs (Ankley et al. 2010).

An important asset of our reporter systems is the temporal information on the activation of cellular defense programs after toxicological insult. This allows the definition of a detailed stress-response fingerprint for individual compounds. Since our method also marks the onset of cell death, this would include the identification of a point-of-no-return or tipping point, reflecting both the concentration and time point after which a certain fraction of cells dies because the defensive programs cannot cope with the level of stress induced by the toxicant. Together, the activation of certain adaptive stress response programs, the order of activation of these programs, the concentration or time after which the tipping point is reached will be of great benefit for risk assessment early in the toxicity testing pipeline and for realization of more mechanistically defined AOPs.

An important feature of our reporter cell systems is that we can detect DILI compound stress responses that are observed in primary human hepatocytes. For a proof of concept, we concentrated on four downstream target genes for oxidative stress (Srxn1), UPR (BiP, Chop) and DDR (p21). We observed a strong concordance for in particular Srxn1-GFP and p21-GFP reporters and a reduced concordance for the BiP-GFP and Chop-GFP reporters. This suggests that our HepG2 reporter models translate well to responses in PHH. This is in particular of interest since the PHH responses were based on transcriptomics and not protein expression. Our finding would be in agreement with recent observations that the onset of cytotoxicity caused by a broad set of DILI compounds is comparable between HepG2 and PHH (Park and Goldring, personal communication). Discrepancies between PHH and HepG2 reporters could be due to this difference, since it is established that the correlation between transcriptomics and proteomics in the same model does not correlate well. Alternatively, ADME and/or cell physiological differences between HepG2 reporters and PHH could explain the differences. The Srxn1-GFP reporter showed the highest concordance with PHH, also suggesting a conservation of the Keap1/Nrf2/Srxn1 pathway activation in HepG2 cells compared to PHH.

We have established our reporters in HepG2 cells. The adaptive stress response pathways that we have incorporated in these cells are not specific to liver cells and involved in the regulation of toxicity in most if not all cells in the body, albeit most likely with different set points. As such, our HepG2 reporters could be representative for general toxicity. Induced pluripotent stem cell technology in combination with genetic recombineering strategies will allow the integration of the GFP reporters in iPSC followed by the differentiation in any cell type. This would open the way for the assessment of the adaptive stress pathway activation in any differentiated cell type as well as the precise quantitative understanding of the differences in control and activation between the various cell types in a same genetic background.

In conclusion, we established a robust high throughput imaging-based platform for the single-cell assessment of adaptive stress response pathway activation in a temporal fashion. This platform can contribute to a mechanism-based chemical safety assessment in both an industry and regulatory setting.

## Materials and methods

### Reagents

All compound drugs were acquired from Sigma-Aldrich, except for cisplatin (Ebewe), CDDO-Me (kind gift from Dr. Ian Copple, University of Liverpool), bendazac (kind gift from Dr. Anita Dankers, Janssen Pharmaceutics), metformin (MIP DILI consortium), propylthiouracil, captopril, tacrine, thioridazine, azathioprine and sulindac (all a kind gift from Dr. Weida Tong, NCTR-FDA). All compounds were freshly dissolved in DMSO, except for metformin, venlafaxine, methapyrilene, fluphenazine, buthionine sulfoximine, bromoethylamine, lomustine (all PBS), acetaminophen, 2,4-dinitrophenol and phenobarbital (all DMEM).

### Cell culture

Human hepatoma HepG2 cells were acquired from ATCC (clone HB8065) and maintained and exposed to drugs in DMEM high glucose supplemented with 10 % (v/v) FBS, 25U/mL penicillin and 25 μg/mL streptomycin. The cells were used between passage 5 and 20. For live cell imaging, the cells were seeded in Greiner black μ-clear 384 wells plates, at 20,000 cells per well.

### Generation of GFP-tagged cell lines

Human *KEAP1*, *NFE2L2* (Nrf2), *CDKN1A* (p21), *TP53* (p53), *BTG2*, *TP53BP1* (53bp1), *XBP1*, *DDIT3* (Chop), *ATF4*, *HSPA5* (BiP) and mouse *SRXN1* BAC clones were selected and GFP-tagged as described previously (Poser et al. 2008b) and stably introduced into HepG2 cells by transfection and 500 μg/ml G-418 selection. At least 20 of the monoclonal BAC-transfected HepG2 colonies were separately grown out, and GFP-positive clones suitable for imaging were selected to complement the BAC-GFP stress response reporter platform.

### RNA interference

siRNAs against human *NFE2L2* (NRF2), *TP53* (P53), *ATF4*, *ATF6* and *EIF2AK3* (PERK) were acquired from Dharmacon (Thermo Fisher Scientific) as siGENOME SMARTpool reagents, as well as in the form of four individual siRNAs. HepG2 cells were transiently transfected with the siRNAs (50 nM) using INTERFERin (Polyplus) as described previously (Fredriksson et al. 2011).

### Western blotting

Samples were collected by direct cell lysis (including pelleted apoptotic cells) in 1× sample buffer supplemented with 5 % v/v β-mercaptoethanol and heat-denatured at 95 °C for 10 min. The separated proteins were blotted onto PVDF membranes before antibody incubation in 1 % BSA in TBS-Tween20. Antibodies: mouse-anti-GFP (Roche) and mouse-anti-tubulin (Sigma) and mouse-anti-GAPDH (Santa Cruz), all antibodies were diluted 1000 times. Horseradish peroxidase detection was performed by Pierce^®^ ECL (Thermo Scientific) using the ImageQuant LAS4000 (GE HealthCare). Cy5 was detected by the ImageQuant LAS4000 (GE HealthCare).

### Microscopy

Accumulation of target protein-GFP levels, localization or foci formation and propidium iodide staining was monitored using a Nikon TiE2000 confocal laser microscope (lasers: 561, 488 and 408 nm), equipped with an automated stage and perfect focus system. Prior to imaging at 20× magnification and either 1X, 2X or 4X zoom, HepG2 cells were loaded for 45 min with 100 ng/mL Hoechst 33342 to visualize the nuclei, upon which the Hoechst-containing medium was washed away to avoid Hoechst phototoxicity (Purschke et al. 2010). After Hoechst-33342 staining, compound exposure was conducted, followed by automated 24-h live cell confocal imaging. The time interval was dependent on the required resolution for the corresponding reporter cell line and on the number of reporter types plated simultaneously on the imaging plates. Cell death was determined by monitoring the accumulation of PI stained cells after a 24-h time period.

### Quantitative image analysis

Image quantification was performed with CellProfiler version 2.1.1 (Kamentsky et al. 2011) with an in-house developed module implementing the watershed masked algorithm for segmentation (Di et al. 2012). The watershed separates an image in regions with single cells followed by pixel classification for each region as fore- or background, and this method performs well detecting the Hoechst_33342_ stained nuclei of the closely packed HepG2 cells. The binary mask containing the segmented nuclei was fed to the *identify*-*primary*-*objects module*, *overlap*-*based*-*tracking* module and *intensity*-*nuclei*-*size*-*shape*-*measurement* modules of CellProfiler. For the cytosol location of the Srxn1-GFP, Btg2-GFP and BiP-GFP reporters, the nuclear objects were used as seeds for the *identify*-*secondary*-*objects* module set to a propagation method with the MCT algorithm on adaptive (window size approximately 20 pixels) segmentation. The Keap1-GFP and 53bp1-GFP reporters are based on foci detection. The nuclei are segmented and used as seeds for the cytosol identification using the cytosolic GFP signal for the Keap1-GFP cell line. The foci detection is performed with the FociPicker3D plug-in (Du et al. 2011) in ImageJ, and each individual focus is assigned to either the nuclei (for 53bp1) or cytosol (Keap1) using the CellProfiler assign parent–child relationship module. The p21, p53, Nrf2, Xbp1, Atf4 and Chop reporters are based on quantifying the GFP signal in the nuclei. The nuclei signal is segmented, and these regions are directly used to quantify the GFP intensity. Segmentation results were stored as PNG files for quality control purposes, and CellProfiler pipelines were stored for reproducibility. Image analysis results were stored on the local machine as HDF5 files.

### Data analysis

Data analysis, quality control and graphics were performed using the in-house developed R package H5CellProfiler (Wink, manuscript in preparation). The features of interest were extracted from the HDF5 files and further analyzed using the graphical user interface of the H5CellProfiler package. The mean of single-cell features for each compound, concentration, cell line and replicate combination was calculated. To account for PI background staining and noise, the segmented PI segmentations were masked by a 2 pixel dilated nuclei. The area of these nuclei and the PI were divided to obtain the cell death stain to cell area ratio. These ratios were filtered to be at least 10 % of the cell size, and following this procedure, each cell was either flagged as alive or dead in the final time point of the 24 live imaging sessions. In this manner, the fraction of dead cells could be accurately determined. All resultant summarized data were stored as tab-delimited text files and further processed for normalization and graphical presentation using R.

### Scaling and plate normalization

For model compound dataset, the different imaging measures were standardized by scaling (i.e., mean or sum of intensity measures, foci counts), and scaling was used for each plate—cell line combination:

$$ x_{\text{scaled}} = \frac{{x - x_{ \hbox{min} } }}{{x_{ \hbox{max} } - x_{ \hbox{min} } }} $$

For the DILI compound dataset, the fraction of GFP-positive cells was determined to increase sensitivity of the assay. A GFP-positive cell was defined as minimally twice the DMSO—control background level. No scaling was used for the GFP-positive fraction measures. The total imaging time and time intervals for the different plates and replicates varied; thus, for statistical analysis and plate normalization regression was performed using the ‘lm’ function of the ‘stats’ package. Natural spline regression with 6 degrees of freedom was performed using the ‘ns’ function of the ‘splines’ package. The additional linear constraints of the natural spline algorithm at the predictor boundaries allowed a stable extrapolation of the total time to equal length for all plates. Twenty-four equidistant time points for each condition were sampled from the model and subjected to quantile normalization to equalize the distributions for each plate.

### Statistical analysis

The quantile normalized data were subjected to statistical significance tests with the following set of formulae; the mean over the replicates for the DMSO controls for each reporter rp and time point tp:

$$ \bar{x}_{\text{DMSO}} \left( {{\text{rp}},{\text{tp}}} \right) = \frac{1}{\text{repl}}\mathop \sum \limits_{r = 1}^{\text{repl}} x_{\text{DMSO}} \left( {{\text{rp}}, {\text{tp}}} \right) $$

The difference *x*_diff between the DMSO means and treatments normalized with standard errors at each reporter rp, treatment tr and time point tp. With the standard errors:

$$ x_{\text{diff}} \left( {{\text{repl}}, {\text{rp}}, {\text{tr}}, {\text{tp}}} \right) = \frac{{x - \bar{x}_{\text{DMSO}} }}{{\sqrt {\sigma_{\text{DMSO}}^{2} + \sigma_{{{\text{DMSO}}, {\text{resid}}}}^{2} + \sigma_{\text{tr}}^{2} + \sigma_{{{\text{tr}}, {\text{resid}}}}^{2} } }} $$

The standard error over the replicates of the DMSO controls, for each reporter and time point:

$$ \sigma_{{x_{\text{DMSO}} }} \left( {{\text{rp}}, {\text{tp}}} \right) = {\text{sd}}\left( {x_{\text{DMSO}} \left( {{\text{rp}}, {\text{tp}}} \right)  } \right) $$

The standard error over the replicates, for each reporter, treatment and time point:

$$ \sigma _{x} \left( {{\text{rp}},\;{\text{tr}},\;{\text{tp}}} \right) = {\text{sd}}\left( {x\left( {{\text{rp}},\;{\text{tr}},\;{\text{tp}}} \right)} \right) $$

The mean residual standard error, with resid the residuals from the regression, for each replicate, reporter and treatment. This ensures the variance from the raw data is included in the statistical analysis.

$$ \sigma_{{\hat{x},{\text{resid}}}} \left( {   {\text{repl}}, {\text{rp}}, {\text{tr}}} \right) = \sqrt {\left( {\frac{{\sum {{\text{tp }}({\text{resid}}\left( {   {\text{repl}}, {\text{rp}}, {\text{tr }}} \right))^{2} } }}{{df_{\text{tp}} - 1}}} \right)} $$

Finally, for each replicate, reporter and treatment, the mean difference over time meanDiff is calculated:

$$ x_{\text{meanDiff}} \left( {   {\text{repl}}, {\text{rp}}, {\text{tr}}} \right) = \frac{1}{\text{tp}}\mathop \sum \limits_{{{\text{tp}} = 1}}^{\text{tp}} x\left( {   {\text{repl}}, {\text{rp}}, {\text{tr}}} \right) $$

A two-sample one-sided Student’s *t* tests between the meanDiff values of matched DMSO control replicates and treatment replicates were performed. A one-sided test was chosen as we are only interested in positive responses with respect to our DMSO controls. To control for the *p* value gained by using a one-sided test, all *p* values were multiplied by 2.

In summary, the average and standard error for each point in time of the quantile normalized values were calculated over the replicates for DMSO. The distance of the treatments to the DMSO mean at each point was determined and normalized by four standard error terms; the standard error of the treatment replicates, the DMSO replicates and the mean residual standard error of the regression analysis for the treatments and controls. The mean difference over time was calculated, followed by a one-sided Student’s *t* test to determine whether the replicate treatment curves were significantly different in the positive direction compared to the DMSO control replicates.

### Cluster analysis

Cluster analysis was performed using the ‘dist’ and ‘hclust’ functions from the ‘stats’ package from the base R distribution. For all cluster analysis, the distance metric was ‘euclidean’ and clustering algorithm ‘complete.’ The clustering of the time curve data required clustering of an extra dimension (time). All pairwise time curve distances were computed. The mean distances per compound–compound and reporter–reporter combinations were calculated, reduced the dimensions to 2 and used as input for the clustering algorithm.

### Data representation

All HCI data representations were generated or modified with Illustrator CS6, Fiji, ggplot2 (Wickham 2009), the aheatmap function of the NMF package (Gaujoux and Seoighe 2010). For response data clustering, the equidistant sample time profile features from the b-spline model were used to calculate a distance matrix for each feature separately using Euclidean distance. A mean distances matrix was calculated and subjected to clustering with the ward.D method of the hclust function.

### Gene expression analysis

CEL files were downloaded from the Open TG-GATEs database: ‘Toxicogenomics Project and Toxicogenomics Informatics Project under CC Attribution-Share Alike 2.1 Japan’ http://dbarchive.biosciencedbc.jp/en/open-tggates/desc.html. Probe annotation was performed using the hthgu133pluspmhsentrezg.db package version 17.1.0, and Probe mapping was performed with hthgu133pluspmhsentrezgcdf downloaded from NuGO (http://nmg-r.bioinformatics.nl/NuGO_R.html). Probe-wise background correction (Robust Multi-Array Average expression measure), between-array normalization within each treatment group (quantile normalization) and probe set summaries (median polish algorithm) were calculated with the rma function of the Affy package (Affy package, version 1.38.1 (Irizarry et al. 2003). The normalized data were statistically analyzed for differential gene expression using a linear model with coefficients for each experimental group within a treatment group. (Smyth 2004; Wolfinger et al. 2001). A contrast analysis was applied to compare each exposure with the corresponding vehicle control. For hypothesis testing, the empirical Bayes statistics for differential expression was used followed by an implementation of the multiple testing correction of Benjamini and Hochberg (1995) using the LIMMA package (Smyth 2004).

## Electronic supplementary material

Below is the link to the electronic supplementary material.

Supplemental Fig. 1 Cytotoxicity measurements after exposure to reference compounds. The percentage of dead cells was determined by the analysis of the overall Hoechst 33342 positive nuclei in an image that was positive for propidium iodide (PI). The fraction of PI-positive cells for all compounds dose combinations for each individual reporter cell line is shown. (Bars indicate concentrations: lowest = red; middle = green; highest = blue). The number of cells after the overnight imaging session was determined by cell counting Hoechst 33342 positive cells, as the average per image for that compound dose combinations. (EPS 1351 kb)

Supplemental Fig. 2 Time course responses of OSR reporter SRXN1, UPR reporters Chop (*DDIT3*) & BiP (*HSPA5*) and DDR reporter p21 (*CDKN1A*) of top 10 selected TG-GATES PHH compounds. Compounds (rows) and reporters (columns) are ordered alphabetically. Reported responses are average of three replicates. Significance is depicted as * = p < 0.05, ** = p < 0.01 and *** = p < 0.001. (EPS 4958 kb)

Supplemental Table 1 Characteristics reference compounds. Table with the selected references compounds, their mechanism of action and the adaptive stress response which are induced by the reference compounds. (EPS 1056 kb)

## Abbreviations

ADR: Adverse drug reaction
AOP: Adverse outcome pathway
BAC: Bacterial artificial chromosome
BFA: Brefeldin A
CDDO-Me: Bardoxolone methyl (methyl-2-cyano 3,12-dioxooleano-1,9-dien-28-oate)
DDR: DNA damage response
DEM: Diethylmaleate
DILI: Drug-induced liver injury
ER-stress: Endoplasmic reticulum stress
IAA: Iodoacetamide
OSR: Oxidative stress response/antioxidant pathways
UPR: Unfolded protein response
Tc: Tunicamycin
Tg: Thapsigargin
